# Donor Preconditioning with Inhaled Sevoflurane Mitigates the Effects of Ischemia-Reperfusion Injury in a Swine Model of Lung Transplantation

**DOI:** 10.1155/2021/6625955

**Published:** 2021-01-08

**Authors:** Alessandro Bertani, Vitale Miceli, Lavinia De Monte, Giovanna Occhipinti, Valeria Pagano, Rosa Liotta, Ester Badami, Fabio Tuzzolino, Antonio Arcadipane

**Affiliations:** ^1^Division of Thoracic Surgery and Lung Transplantation, Department for the Treatment and Study of Cardiothoracic Diseases and Cardiothoracic Transplantation, IRCCS-ISMETT, Palermo, Italy; ^2^Research Department, IRCCS-ISMETT, Palermo, Italy; ^3^Department of Anesthesiology and Critical Care, IRCCS-ISMETT, Palermo, Italy; ^4^Fondazione Ri.MED, Palermo, Italy; ^5^Department of Pathology, IRCCS-ISMETT, Palermo, Italy; ^6^Department of Laboratory Medicine and Advanced Biotechnologies, IRCCS-ISMETT, Palermo, Italy

## Abstract

Primary graft dysfunction (PGD) and ischemia-reperfusion injury (IRI) occur in up to 30% of patients undergoing lung transplantation and may impact on the clinical outcome. Several strategies for the prevention and treatment of PGD have been proposed, but with limited use in clinical practice. In this study, we investigate the potential application of sevoflurane (SEV) preconditioning to mitigate IRI after lung transplantation. The study included two groups of swines (preconditioned and not preconditioned with SEV) undergoing left lung transplantation after 24-hour of cold ischemia. Recipients' data was collected for 6 hours after reperfusion. Outcome analysis included assessment of ventilatory, hemodynamic, and hemogasanalytic parameters, evaluation of cellularity and cytokines in BAL samples, and histological analysis of tissue samples. Hemogasanalytic, hemodynamic, and respiratory parameters were significantly favorable, and the histological score showed less inflammatory and fibrotic injury in animals receiving SEV treatment. BAL cellular and cytokine profiling showed an anti-inflammatory pattern in animals receiving SEV compared to controls. In a swine model of lung transplantation after prolonged cold ischemia, SEV showed to mitigate the adverse effects of ischemia/reperfusion and to improve animal survival. Given the low cost and easy applicability, the administration of SEV in lung donors may be more extensively explored in clinical practice.

## 1. Introduction

Primary graft dysfunction (PGD) presents as acute lung injury (ALI) in up to 10-30% of patients undergoing lung transplantation (LTx), mostly in the first 72 postoperative hours [1]. Its pathogenesis has been related to ischemia-reperfusion injury (IRI) [2]. Severe PGD may adversely impact on the long-term outcomes of LTx and is associated with the development of chronic lung allograft dysfunction (CLAD) [3, 4].

Different studies, aiming at understanding the molecular and cellular mechanisms of PGD, have highlighted different unmodifiable and modifiable risk factors. Among unmodifiable factors, specific polymorphisms in several immunomodulatory genes have shown association with an increased risk of PGD in lung transplant recipients [2]. On the other hand, among modifiable factors, donor and recipient features and technical factors can influence the occurrence of PGD [5].

Several strategies have been investigated to prevent and treat PGD, to quote a few, the use of low potassium dextran (LPD) solutions for perfusion [6], the use of inhaled nitric oxide [7], exogenous surfactant [8], carbon monoxide [9], and platelet-activating factors [10]. Although many of these treatments have shown significant beneficial effects, only a few have been integrated into clinical practice due to the high cost or potential adverse effects.

Increasing evidence shows that the administration of volatile anesthetics may offer protection against organ ischemic damage through a mechanism called anesthetic conditioning (AC) and attenuate the IRI process [11, 12]. Based on the timing of administration, AC is defined as preconditioning (before ischemia), perconditioning (during ischemia), or postconditioning (after reperfusion). Anesthetic preconditioning (APC) with volatile anesthetics like sevoflurane, halothane, and isoflurane has shown to reduce IRI damage in different experimental models [13]. Sevoflurane (SEV) is one of the most commonly used volatile anesthetic agents, and the effects of SEV preconditioning and postconditioning have been well investigated in the setting of cardiac ischemia. SEV reduces the size of myocardial infarction by activating *K* (ATP) channels and reduces the time threshold for ischemic preconditioning in experimental dog models of *in vivo* cardiac ischemia [14]. A randomized clinical study has also shown that SEV can have a protective role on late cardiac events in individuals undergoing coronary artery bypass surgery [15].

In LTx, preconditioning of lungs with inhaled SEV has been associated with reduced IRI injury in *ex vivo* models of isolated rat lungs [16], in an *in vivo* model of rat pulmonary and hepatic injury [17], and in an *in vivo* model of swine autotransplantation [18]. Kalb et al. showed that preconditioning but not postconditioning with SEV has beneficial effects in a rat IRI model [19]. Furthermore, both preconditioning and postconditioning with SEV have shown beneficial effects in a rat LTx model of cold ischemia [20]. In summary, APC with SEV has shown to mitigate IRI damage in small animal studies, but, as far as we know, a limited number of studies are available in the *in vivo* LTx large animal model.

In this study, we tested the effects of donor preconditioning with SEV in a swine model of single LTx after prolonged (24 hours) cold ischemic graft preservation. The hypothesis of the study was that donor SEV administration could mitigate the negative effects of ischemia-reperfusion injury.

## 2. Materials and Methods

The study was approved by the IRRB (Institutional Research Reviewer Board) and by the OPBA (Institutional Animal Welfare and Protection Agency). All experiments were performed according to the Italian and European guidelines on animal welfare.

### 2.1. Animal Model and Design of the Study

**Figure 1:**
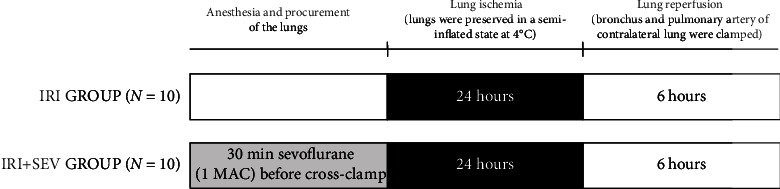
Experimental design. In the IRI group, the left lung was preserved in a semi-inflated state at 4°C for 24 hours and then implanted in the animal recipients, which were then observed and monitored for 6 hours. In the IRI+SEV group, animal donors received pretreatment with SEV for 30 min prior to cross-clamp and perfusion. Then, the graft was preserved in a semi-inflated state at 4°C for 24 hours and implanted in the animal recipients, which were then observed and monitored for 6 hours. SEV: sevoflurane; IRI: ischemia-reperfusion injury.

Twenty hybrid Golan domestic pigs, weighting between 25 and 35 kg, underwent single left LTx from donor pigs of similar weight range after 24 hours of cold graft preservation. Donor animals were block randomized to receive inhalation of sevoflurane (SEV group, *n* = 10) or no inhalation of anesthetic gas (control group, *n* = 10) (Figure 1).

### 2.2. Anesthesia

All animals were kept on a clear liquid diet for 24 hours before each experiment. Premedication was performed with an intramuscular injection of atropine (0.025 mg/kg) and zolazepam/tiletamine (5 mg/kg). After monitoring pulse-oximetry and electrocardiography, general anesthesia was induced with thiopental (6 mg/kg) and fentanyl (3 μg/kg), and the animals were intubated in a prone position with a #7 single-lumen orotracheal tube. Mechanical ventilation was initiated on a volume-control mode (tidal volume of 7 mg/kg), with 5 cm H_2_O positive end-expiratory pressure (PEEP), a fraction of inspired oxygen (FiO_2_) of 0.5, and a respiratory rate of 14. Anesthesia was maintained with an i.v. continuous infusion of propofol (100 μg/kg/min), ketamine (10 mg/kg/min), and fentanyl (45 μg/kg/h). Muscle paralysis was achieved with a cisatracurium bolus (0.2 mg/kg) and continuous infusion (0.06 mg/kg/h). Throughout the procedure, an i.v. infusion of 5% glucose and crystalloids was administered at the rate of 1 ml/kg/h. In all recipient animals, a continuous infusion of epinephrine (0.01 μg/kg/min) was administered after reperfusion of the transplanted lung in order to prevent hemodynamic impairment. At the end of each experiment, all animals were euthanized under deep anesthesia using an i.v. administration of Tanax (embutramide, mebezonius, tetracaine, 10 mg/kg). Donor animals in the SEV group were given sevoflurane (Baxter, Deerfield IL, US) through the ET tube for 30 minutes before cross-clamp and perfusion, without any washout, with a concentration of 1 MAC.

### 2.3. Surgical Protocol

#### 2.3.1. Donors

After induction of anesthesia, the donor animals were placed in the supine position, scrubbed with iodine, and prepped with surgical drapes. After median sternotomy, the thymus was excised, and the pericardium opened. The superior vena cava, inferior vena cava, and aorta were encircled with a heavy tie. After full heparinization (300 IUkg), the aorta was clamped, the IVC and left atrial appendage vented and the lungs were flushed with 1 l of Perfadex (XVIVO, Sweden) supplemented with 1 ml of thamesol and 10 mg of epoprostenol. The chest cavity was filled with ice slush, and ventilation was reduced to a RR of 4 and a TV of 1 ml/kg with a FiO_2_ of 50%. The heart was excised before the procurement of the double lung block, leaving a large amount of left atrial cuff, and the double lung block was preserved in a semi-inflated state at 4°C for 24 hours.

#### 2.3.2. Recipients

After induction of anesthesia, a Swan-Ganz catheter (Edwards) and an arterial line were placed in the right internal jugular vein and carotid artery, using a cut-down technique. Through a left thoracotomy in the 4^th^ intercostal space, a left intrapericardial pneumonectomy was performed leaving an adequate cuff of bronchus, pulmonary artery, and veins. A single bolus of 1000 UI of heparin was administered at this time. The left graft was prepared on the back table and implanted in the recipient (using the following sequence: bronchus, left atrium, and pulmonary artery) using 4-0 and 5-0 Prolene running sutures. The graft was slowly reperfused in a retrograde and anterograde fashion, and adequate deairing was performed before securing the sutures. Approximately 10 minutes after reperfusion, the contralateral bronchus and pulmonary artery were clamped to exclude any physiological contribution of the native right lung. The animals were then observed and monitored for 6 hours before receiving euthanasia.

### 2.4. Monitoring and Observation Phase

Donors underwent noninvasive monitoring of hemodynamic and respiratory parameters throughout the procedure and received an ABG, a bronchoalveolar lavage (BAL), and a right lung biopsy. Recipients underwent ABGs and recording of hemodynamic and ventilatory parameters after induction of anesthesia, 15 minutes before and 15 minutes after reperfusion of the graft, and to follow, every 30 minutes for 6 hours.

### 2.5. Ventilatory Measurements

Peak (PIP) and plateau (PlatP) respiratory pressures were recorded with the ventilator set at stable settings (TV = 7 ml/kg, RR = 14, FiO_2_ = 50%). Static compliance was calculated using the Cstat = VT/(PlatP − PEEP) formula. Arterial blood gases were drawn from the invasive arterial line and included the measurement of pH, peripheral saturation (SpO_2_), partial pressure of oxygen (PaO_2_), PaO_2_/FiO_2_ ratio, partial pressure of carbon dioxide (PaCO_2_), base excess (BE), lactates (LAC), and hematocrit (Ht).

### 2.6. Hemodynamic Measurements

Heart rate (HR), mean arterial pressure (MAP), central venous pressure (CVP), pulmonary arterial pressure (PAP), and pulmonary capillary wedge pressure (PCWP) were monitored continuously and recorded in the recipient at all the above described reference time points. Cardiac output (CO) and cardiac index (CI) were also continuously recorded through the PA catheter using the thermodilution technique and a Vigilance apparatus (Edwards, US).

### 2.7. Histology and Pathologic Grading

Surgical lung biopsies were performed using a linear 45 mm stapler before cross-clamp (baseline) and after cold preservation (postischemic) in the donor right lung, to avoid unnecessary damage to the left lung graft. A further biopsy of the left (transplanted) lung was performed after completion of the observation period. Biopsies were fixed in 4% phosphate-buffered formalin, cut, and embedded in paraffin. 4 *μ*m sections were cut and stained with hematoxylin and eosin (H&E). Alveolar edema, interstitial edema, alveolar neutrophil infiltration, perivascular neutrophil infiltrate, interstitial hemorrhage, fibrin and hyaline deposits, chronic infiltrate, and dense fibrosis were graded on a scale between 0 (minimal) and 3 (severe). A semiquantitative histological evaluation score was used to assess the morphological changes in the specimens. Each item was assigned a score by two independent pathologists, and a histological total lung injury score per slide was calculated.

### 2.8. Bronchoalveolar Lavage

A BAL was performed using a fiberoptic bronchoscope in the donor right lung (after induction of anesthesia) and in the recipient left transplanted lung (2 and 6 hours after reperfusion). 120 ml of saline solution were injected in a subsegmental bronchial branch and recollected under direct visualization using low-pressure suction. BAL samples were centrifugated at 150 × g for 10 min, and the supernatant was preserved at -80°C. An ELISA kit for cytokines was used to assess the expression of IL-12, IL-10, IL-8, IL-6, IL1*β*, TNF*α* (Thermo Scientific, USA), and TGF-*β* (MyBioSource, USA).

### 2.9. Statistical Analysis

Standard descriptive statistics were used to present categorical and continuous data, as appropriate. One-way ANOVA analysis of variance or, in case of a negative Bartlett test, the Kruskal-Wallis model was used to compare means between groups. Survival of animals after transplantation was compared between groups using the Kaplan-Meier model. Repeated measures were analyzed using ANOVA, ANCOVA, and GEE (generalized estimating equation). Data expressed as median (25%-75% quartiles) were tested using Student's *t*-test or Mann–Whitney *U* test when appropriate. Random effect and Robust Regression models are used in order to describe not only numerical differences between each group (“group effect” measures) but also differences across time within the same group (“time effect” measures) and, more importantly, the combined effect of the two observations (“interaction effect” measures). The Chi-square and Stuart-Maxwell models were used to analyze the results of the pathological review.

## 3. Results

### 3.1. Baseline Characteristics and Survival

**Figure 2:**
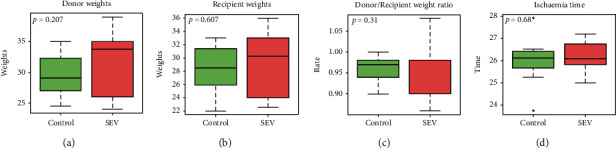
Baseline measurements of weight of donors (a), weight of recipients (b), donor recipient weight ratio (c), and ischemia time (d) in the control group and sevoflurane (SEV) group. The circle is an indicator of the position that is off average. Data are expressed as median (quartile 25%-quartile 75%).

**Figure 3:**
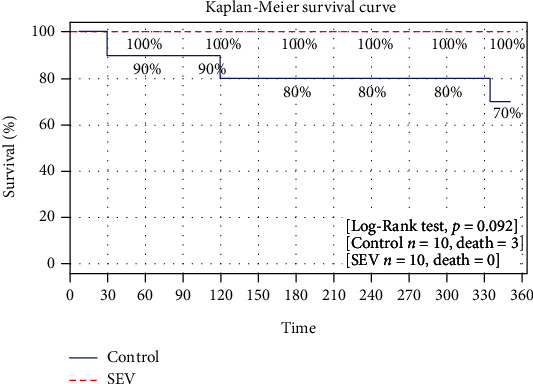
Kaplan–Meier curves of recipient's survival during the six hours of observation in the control group and sevoflurane (SEV) group. Survival was tested with the log-rank test.

Donor weight was slightly higher than recipient weight within both groups (in average, 30.7 kg and 28.7 kg, respectively) (Figures 2(a) and 2(b)), although not statistically significant between each group (Figure 2(c)). Cold ischemia time was also similar between the two groups (control, 26.2 hrs and SEV 26.0 hrs) (Figure 2(d)). All recipients in the SEV group survived for the entire length of the observation period, while three recipients in the control group died before the completion of the 6-hour observation period (after 30 min, 2 hrs, and 5 h 30 min, respectively) (Figure 3), because of severe pulmonary edema and right heart failure.

### 3.2. Lung Function Analysis

Table 1 shows the association between ventilatory and hemodynamic parameter variations over time and between the control and SEV groups.

**Figure 4:**
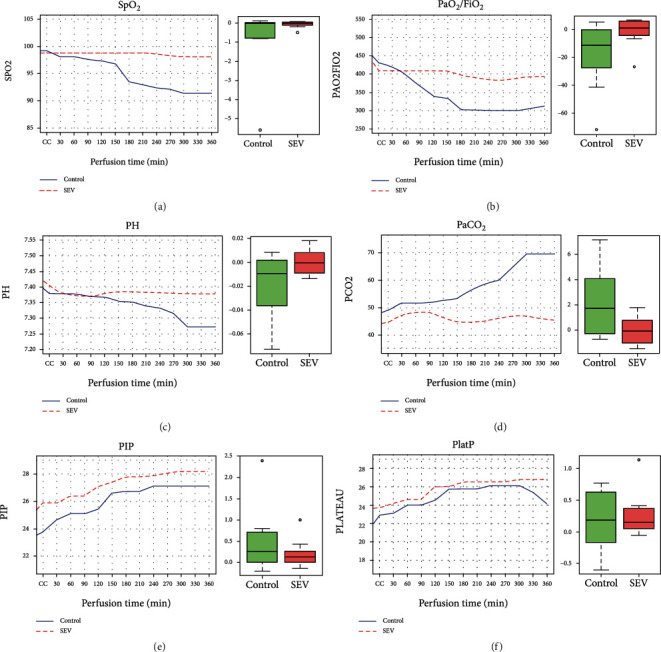
Pulmonary physiological variables detected during the observation period in the control group and sevoflurane (SEV) group. In the left panel, the data are presented as median from GEE modeling. In the right panel, the data are presented as median of quartile 25%-quartile 75%, and the circle is an indicator of the position that is off average. (a) SpO_2_ (peripheral saturation); (b) PaO_2_/FiO_2_ (partial pressure of oxygen/FiO_2_ ratio); (c) pH; (d) PaCO_2_ (partial pressure of carbon dioxide); (e) PIP (peak inspiratory pressure); (f) PlatP (plateau pressure); CC: contralateral clamp.

SpO_2_, the PaO_2_/FiO_2_ ratio, and the pH tended to remain stable along the observation period in the SEV group, while progressively decreased in the control group. In detail, the levels of SpO_2_ (Figure 4(a)), PaO2/FiO2 (Figure 4(b)), and the pH (Figure 4(c)) decreased significantly in the control group. Conversely, the PaCO_2_ of the control group increased, while remained stable in the SEV group (Figure 4(d)). The PIP and PlatP pressures showed a significant increase (*p* value < 0.01 within the group) in both groups, although no significant differences were found between the two groups (Figures 4(e) and 4(f)).

**Figure 5:**
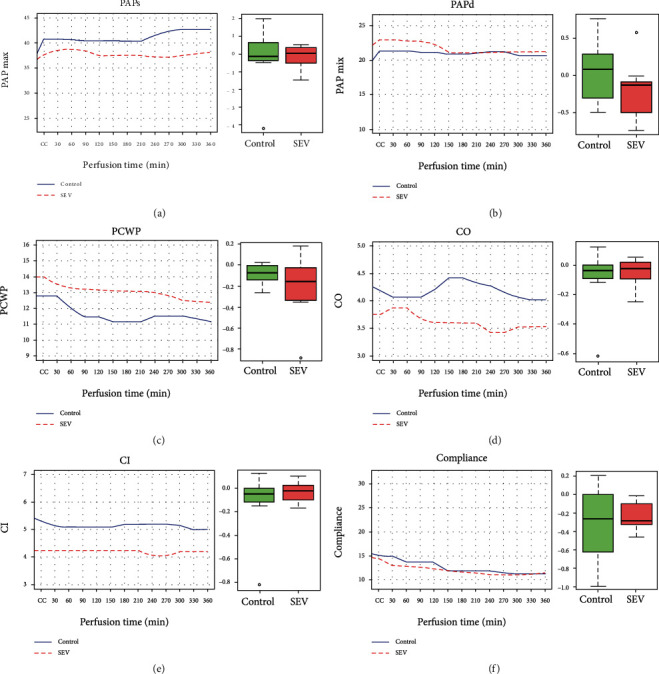
Hemodynamic physiological variables detected during the observation period in the control group and sevoflurane (SEV) group. In the left panel, the data are presented as median from GEE modeling; in the right panel, the data are presented as median of quartile 25%-quartile 75%, and the circle is an indicator of the position that is off average. (a) PAPs (systolic pulmonary arterial pressure); (b) PAPd (diastolic pulmonary arterial pressure); (c) PCWP (pulmonary capillary wedge pressure); (d) CO (cardiac output); (e) CI (cardiac index); (f) compliance. CC: contralateral clamp.

Both systolic and diastolic PAP as expected rose after contralateral cross-clamp. During the 6-hour observation period, the PAP showed minimal, nonsignificant changes within both groups (Figures 5(a) and 5(b)). The PCWP, the CO, and the CI showed minimally significant decreasing values within each group without any significant differences between the two arms (Figures 5(c)–5(e)). The static pulmonary compliance slope decreased significantly over time in both groups, and the difference between the two groups was not significant (Figure 5(f)).

We also detected the values of other pulmonary physiological variables such as lactate, base excess, hematocrit, mean arterial pressure, heart rate, and central venous pressure, and the results are summarized in Supplementary Figure 1.

### 3.3. Lung Histology

**Figure 6:**
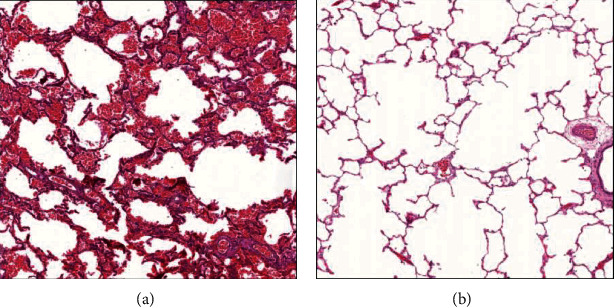
Representative H&E pathology of LTx: (a) grafts in the control group showed typical pathology of PGD. (b) Grafts in the SEV group showed significantly attenuated PGD pathology when compared with controls.

Histologically, transplanted lungs in the control group (*n* = 10) showed severe traits of PGD, characterized by diffuse alveolar septal thickening, edema, and neutrophilic infiltrates (Figure 6(a)). On the other hand, the SEV group (*n* = 10) showed significant less damage of PGD pathology (Figure 6(b)).

We analyzed the pulmonary injury scores in the donor before cross-clamp (baseline), immediately before transplantation on the back table (IRI injury), and after transplantation, at the end of the observation period in both the control group (*n* = 10) and SEV group (*n* = 10). The semiquantitative evaluation revealed a significant difference in the degree of pulmonary injury between the two groups. In detail, before transplantation (back table), lung injury in controls showed increased severity of both alveolar neutrophil infiltration and perivascular neutrophil infiltrate when compared with the SEV group. Furthermore, the increase of alveolar edema was observed after transplantation (postreperfusion) in the control group compared to the SEV group. The total lung injury score, both before and after transplantation, showed an increased value of lung injury in the control group if compared with the SEV group (Table 2).

### 3.4. Expression of Cytokines in BAL

We performed an analysis of cytokines in the BAL of donor lungs (baseline) and in the recipient transplanted lung (both in control group *n* = 10 and SEV group *n* = 10), 2 and 6 hours after reperfusion. We measured IL-12, IL-10, TGF-*β*, TNF*α* IL-8, IL-6, and IL-1*β* as markers of inflammation of the pulmonary parenchyma. As shown in Table 3, the baseline levels were very low for IL-10, TGF-*β*, TNF*α*, IL-6, and IL-1*β*. In both the SEV and the control group, all cytokines increased from the baseline value except for IL-10 in the control group (both after 2 and 6 hours of reperfusion) and TGF-*β* in the SEV group (both after 2 and 6 hours of reperfusion). Furthermore, in the SEV group, the levels of IL-12 (mainly after 6 hours of reperfusion), TNF*α* (both after 2 and 6 hours of reperfusion), IL-8 (both after 2 and 6 hours of reperfusion), IL-6 (mainly after 2 hours of reperfusion), and IL-1*β* (mainly after 2 hours of reperfusion) were lower than in the control group (Table 3).

## 4. Discussion

As shown in a landmark prospective cohort study of 1,255 lung recipients enrolled in the US between 2002 and 2010, the overall incidence of grade 3 primary graft dysfunction (PGD) at any time point in the first 72 hours can be as high as 30.8%. PGD grade 3 is also associated with higher mortality and with the development of bronchiolitis obliterans syndrome (BOS) [1].

Ischemia-reperfusion injury (IRI) is the most relevant pathophysiologic mechanism underlying PGD [2]. IRI is a complex process involving both the generation of oxidative stress and the release of inflammatory cytokines that lead to cell death [21].

It has been shown that anesthetic conditioning (AC) may attenuate the IRI process [11, 12]. AC is safe and easy to perform and is routinely used for anesthesia in surgical patients [22]. SEV is an option for LTx anesthesia in many centers worldwide and, despite contradictory trials in liver and kidney transplantation [23, 24], has shown promising results as a protective treatment for IRI after LTx. Volatile anesthetics may decrease IRI-induced cytokine-mediated lung injury during thoracic procedures, in particular TNF-*α*, IL-1*β*, IL-6, IL-8, and IL-10 [21, 25–27]. Xu et al. showed in a rat model that SEV preconditioning had a protective effect on IRI-induced pulmonary injury, by inhibiting leukocyte recruitment and MMP-9 secretion [17]. Ohsumi et al. suggested that preconditioning or postconditioning of the lungs with SEV can display a protective effect against IRI injury in a rat LTx model. In these studies, SEV significantly improved the oxygenation of lung grafts and reduced pulmonary edema through the reduction of IL-1*β*, IL-6, and TNF-*α*. Moreover, SEV significantly inhibited apoptotic cells by a decrease in cytochrome c release and consequent decrease of caspase-3 cleavage/activation [20]. Garutti et al., in a pig model of lung autotransplantation, confirmed the beneficial effects of anesthetic preconditioning with SEV on intracellular pathways of death [28]. In another mouse LTx model, it has been demonstrated that SEV preconditioning has protective effects on lung grafts by both suppression of inflammatory cytokines and induction of M2 anti-inflammatory macrophages [29]. Wang et al. also suggested that SEV can protect the lungs procured from donors after circulatory death during EVLP in a rat model [16]. Human studies of one-lung ventilation during lung resection have shown that proinflammatory cytokine levels increase in both the ventilated and unventilated lung and that SEV may suppress the alveolar proinflammatory response to a greater extent than propofol [25].

In our study, we aimed to evaluate the effect of donor preconditioning with SEV in a pig LTx model where IRI injury was induced by 24 hours of cold ischemia. In this well-established model of IRI, we decided to clamp the contralateral bronchus and PA in all animals to obtain a reliable assessment of the transplanted lung function and to replicate more closely the clinical setting. Our results showed that treatment with SEV consistently attenuated IRI after LTx by improvingoxygenation and suppressing inflammation. In our work, in addition to monitoring lung physiologic ventilatory and hemodynamic parameters (Table 1), we also investigated conventional markers of acute lung injury after LTx such as pulmonary edema, inflammatory cytokines, and neutrophil activation. We found a significant improvement in both respiratory and hemodynamic parameters of lung grafts in the SEV group. Indeed, during the observation period after LTx, SpO_2_, PaO_2_/FiO_2_, PaCO_2_, and pH remained stable in the SEV group, while a decrease of SpO2, PaO2/FiO2, and pH and an increase of PaCO_2_ were observed in the control group (Figures 4(a)–4(d)). These functional results are reinforced by the improved survival of animals in the SEV group (Figure 3). In our opinion, it is possible that the differences of PAP and Cstat values between groups did not reach statistical significance due to the missing values from control animals that died before the end of the observation period. Moreover, our histological data showed that SEV treatment reduced pulmonary injury (Table 2 and Figure 6). In detail, compared to the SEV group, in the control group, higher levels of inflammation (increased severity of both alveolar neutrophil infiltration and perivascular neutrophil infiltrate) were observed before transplant, and this effect was augmented after transplantation due to the increased alveolar edema in the control group. Furthermore, the total lung injury score, both before and after transplantation, showed an increased value of lung injury in the control group if compared with the SEV group (Table 2). These results highlight the potential anti-inflammatory effects of treatment with SEV.

Different studies suggest that the variation in the levels of some cytokines may correlate with allograft injury in LTx. IL-6 can promote fibrosis by driving chronic inflammation [30] and by activating the TGF*β* pathway [31] that is one of the most potent profibrotic cytokines. It has been shown that there is a correlation between the severity of IRI in PGD and the increased levels of IL-6 [32]. D'Ovidio et al. found that IL-12, a pro-inflammatory cytokine, was increased in the BAL fluid of pateints with acute rejection [33]. On the other hand, IL-10, an anti-inflammatory cytokine, showed beneficial effects on both early and late outcome after LTx [34]. Moreover, evidence from *in vivo* studies suggests that the administration of IL-10 before transplantation improves graft acceptance and survival [35, 36], and IL-10 has been ascribed a protective role against allograft rejection [37, 38].

Interestingly, in our experimental model, we showed that SEV treatment was able to modulate both pro- and anti-inflammatory factors. In particular, we showed that proinflammatory cytokines such as IL-12 (mainly after 6 h reperfusion), TNF*α* (both after 2 and 6 h reperfusion), IL-8 (both after 2 and 6 h reperfusion), IL-1*β* (mainly after 2 h reperfusion), IL-6 (mainly after 2 h reperfusion), and TGF*β* (both after 2 and 6 h reperfusion) were expressed at lower levels in the SEV group than in controls, whereas by contrast, IL-10, an anti-inflammatory cytokine, had higher expression levels in the SEV group than in the control group both after 2 and 6 hours of reperfusion (Table 3). These biochemical data supported our histological data in terms of the anti-inflammatory properties of SEV. Similar results were also obtained by other groups, which showed the efficacy of SEV preconditioning in decreasing inflammatory responses in a pig model of autotransplantation [18].

Based on our knowledge, there are no reports of the use of SEV preconditioning in a heterotransplantation swine model replicating the clinical setting. Our study demonstrates that the SEV preconditioning in a pig LTx model exhibits significant protective effects against IRI by means of anti-inflammatory effects. Although in a rat *in-vivo* model of SEV pre- and postconditioning it has been shown that both strategies provided significant protection against myocardial IRI [39], it is possible that a preischemic conditioning approach may be more applicable to the setting of lung IRI. Indeed, from a practical point of view, we think that the feasibility of a short application of SEV in the organ donor would be more easily applicable in the clinical setting. The use of a unique donor protocol could be rapidly extended to a national level and included in the organ donor management routine as part of a multicentric trial. Also, the effects of the administration of SEV could be beneficial to other organs [40]. The low cost and easy application of this strategy, added to the potential benefit to organs other than the lung, suggest further evaluation and study in clinical trials.

## Figures and Tables

**Table 1:** GEE analysis of association between lung function parameters over time and the control and SEV groups.

Parameters		SEV vs. control	Time	Interaction (control × time)
PaO2/FiO2	Coef.	6.362	-1.379	-12.806
	*p* value	0.915	0.398	<0.001^∗^

pH	Coef.	0.198	-0.001	-0.011
	*p* value	0.578	0.882	<0.001^∗^

PaCO2	Coef.	0.776	-0.061	1.856
	*p* value	0.879	0.774	<0.001^∗^

BE	Coef.	2.295	-0.097	—
	*p* value	0.022^∗^	0.01^∗^	—

Lactate	Coef.	-0.546	-0.013	-0.096
	*p* value	0.55	0.629	0.018^∗^

Ht	Coef.	0.801	0.335	0.358
	*p* value	0.539	0.568	<0.001^∗^

Compliance	Coef.	0.865	-0.233	-0.134
	*p* value	0.682	<0.001^∗^	0.054^∗^

MAP	Coef.	-5.788	-0.951	—
	*p* value	0.326	<0.001^∗^	—

HR	Coef.	-12.739	-0.643	3.536
	*p* value	0.134	0.092	<0.001^∗^

SpO2	Coef.	-0.265	0.061	-0.631
	*p* value	0.912	0.607	<0.001^∗^

PIP	Coef.	-0.674	0.235	—
	*p* value	0.69	<0.001^∗^	—

PlatP	Coef.	-0.012	0.256	—
	*p* value	0.995	<0.001^∗^	—

PAPs	Coef.	1.066	-0.161	0.384
	*p* value	0.693	0.119	0.013^∗^

PAPd	Coef.	-1.727	-0.184	0.236
	*p* value	0.377	0.009^∗^	0.025^∗^

PCWP	Coef.	-1.065	-0.148	—
	*p* value	0.491	<0.001^∗^	—

CO	Coef.	0.305	-0.039	—
	*p* value	0.476	0.002^∗^	—

CI	Coef.	0.709	-0.031	—
	*p* value	0.135	0.030^∗^	—

CVP	Coef.	-2.729	-0.181	0.032
	*p* value	0.034^∗^	0.001^∗^	<0.001^∗^

^∗^(*p* value < 0.05). The *p* value represent the result of GEE model with exchangeable correlation.

**Table 2:** Histological lung injury score.

		Baseline	Back table	Postreperfusion
Alveolar edema	Control	0 (0; 0)	0 (0; 0)	2 (0.25; 2)
	SEV	0 (0; 0)	0 (0; 0)	0,5 (0; 1)^a^

Interstitial edema	Control	0 (0; 0)	0 (0; 0)	1 (0; 2)
	SEV	0 (0; 0)	0 (0; 1)	1 (1; 1.75)

Alveolar neutrophil infiltration	Control	0 (0; 0)	0,5 (0; 1.25)	0,5 (0; 1)
	SEV	0 (0; 0)	0 (0; 0)	1 (0; 1)

Perivascular neutrophil infiltrate	Control	0 (0; 0)	0,5 (0; 1)	1 (0.25; 1)
	SEV	0 (0; 0)	0 (0; 0)^a^	0 (0; 0)^a^

Interstitial hemorrhage	Control	0 (0; 0)	0 (0; 0)	0 (0; 0.75)
	SEV	0 (0; 0.75)	0 (0; 0)	0 (0; 0)

Fibrin and hyaline deposits	Control	0 (0; 0)	0 (0; 0)	0 (0; 0)
	SEV	0 (0; 0)	0 (0; 1)	0 (0; 0.75)

Chronic infiltrate	Control	2 (1; 2)	1,5 (0.75; 2.25)	2 (1; 2)
	SEV	1,5 (1; 2)	2 (1; 2)	2 (1; 2)

Dense fibrosis	Control	0 (0; 0.75)	0 (0; 0.25)	0 (0; 0)
	SEV	0 (0; 0)	0 (0; 0)	0 (0; 0)

Total lung injury score	Control	2 (1; 2.75)	2,5 (0.75; 4.75)	6,5 (1.5; 8.75)
	SEV	1,5 (1; 2.75)	2 (1; 4)	4 (2.25; 6.5)

Control: control groups; SEV: sevoflurane treatment groups. Values are medians and interquartile (25%-75%) range. ^a^*p* < 0.05 vs. control.

**Table 3:** BAL levels of IL-12, IL-10, TGF-*β*, TNF*α*, IL-8, IL-6, and IL-1*β* in animal donor (baseline) and animal recipient after 2 and 6 hours of reperfusion.

		Baseline	2 h reperfusion	6 h reperfusion
IL-12	Control	7.14 (4.6; 12.3)	9.71 (2.7; 41.8)	120.42 (110.6; 152.5)
	SEV	36.25 (0; 93.1)	58.33 (3.7; 98.9)	57.5 (46.0; 80.4)^a^

IL-10	Control	0 (0; 0)	0 (0; 25.3)	0 (0; 0)
	SEV	0 (0; 0)	62.35 (0; 74.8)^a^	17.10 (0; 34.4)^a^

TGF-*β*	Control	0 (0; 0)	43.58 (0; 107.4)	0 (0; 71.2)
	SEV	0 (0; 0)	0 (0; 0)^a^	0 (0; 0)

TNF*α*	Control	0 (0; 0.2)	270.42 (178.8; 414.2)	43.69 (0; 99.2)
	SEV	0 (0; 0)	93.26 (44.7; 191.7)	19.64 (0; 65.5)

IL-8	Control	139.25 (116.1; 146.1)	825.5 (356.7; 1263)	203 (154.2; 384.2)
	SEV	92.7 (0; 204.2)	21.875 (0; 120.6)^a^	137.5 (21.8; 146.2)^a^

IL-6	Control	2.07 (0; 6.5)	93 (52.4; 338.7)	130.30 (77.2; 1032.2)
	SEV	0 (0; 0)	33.88 (0; 69.4)^a^	277.33 (97.7; 420.5)

IL-1*β*	Control	0 (0; 6.5)	255.625 (0; 259.2)	35.5 (11.1; 372.3)
	SEV	0 (0; 14.3)	14.5 (0; 77.5)	168.5 (74.8; 343.1)

Control: control groups; SEV: sevoflurane treatment groups. Values are medians and interquartile (25%-75%) range and are presented as concentration (pg/ml). ^a^*p* < 0.05 vs. control.

## Data Availability

The datasets used or analyzed during the current study are available from the corresponding author on reasonable request.

## Supplementary Materials

Supplementary Materials Supplementary Figure 1: pulmonary physiological variables detected during the observation period in the control group and sevoflurane (SEV) group.
