# Supplementary material for: Donor Preconditioning with Inhaled Sevoflurane Mitigates the Effects of Ischemia-Reperfusion Injury in a Swine Model of Lung Transplantation
Source: Biomed Res Int. 2021 Jan 8;2021:6625955. doi: 10.1155/2021/6625955 (PMC7815409; doi:10.1155/2021/6625955)
Supplement: Supplementary Materials — Supplementary Figure 1: pulmonary physiological variables detected during the observation period in the control group and sevoflurane (SEV) group. [file 6625955.f1.docx]

**Supplementary materials:**

**Supplementary Figure 1:** Pulmonary physiological variables detected during observation period in control group and sevoflurane (SEV) group. In left panel the data are presented as median from GEE modeling; in right panel the data are presented as median of quartile 25% - quartile 75%, and the circle is an indicator of the position that is off average. (a) Lactate; (b) BE (base excess); (c) Ht (hematrocrit); (d) MAP (mean arterial pressure); (e) HR (Heart rate); (f) CVP (central venous pressure). CC: controlateral clamp.
